# Creutzfeldt Cell Rich Glioblastoma: A Diagnostic Dilemma

**DOI:** 10.7759/cureus.1749

**Published:** 2017-10-05

**Authors:** Zain Boghani, William J Steele, Matthew D Cykowski, Leomar Y Ballester, Gavin Britz

**Affiliations:** 1 Department of Neurological Surgery, Houston Methodist Hospital; 2 Department of Pathology and Genomic Medicine, Houston Methodist Hospital

**Keywords:** creutzfeldt cells, glioblastoma, brain tumors, demyelinating disease

## Abstract

Differentiating demyelinating conditions from neoplastic conditions can pose a significant challenge. There are a number of reports in the literature of large ring-enhancing, space-occupying lesions that were initially considered neoplastic or infectious but, on further review, were deemed demyelinating. Creutzfeldt-Peters cells (CPC) are reactive astrocytes with fragmented nuclear inclusions that are routinely seen in multiple sclerosis (MS) and generally exclude the diagnosis of glioblastoma (GB). The patient is a 78-year-old man with a history of prostate cancer, which was treated with radiation therapy, who presented with altered mental status. A magnetic resonance imaging (MRI) scan of the brain revealed a 4.6 x 3.1 cm mass lesion in the right posterior temporal lobe with minimal mass effect and heterogeneous contrast enhancement. The patient underwent an open biopsy of the mass, which on histology was rich with Creutzfeldt-Peters cells. Frozen histology was unclear and full resection of the mass was delayed. A molecular and immunohistochemical analysis confirmed glioblastoma. The patient returned four weeks later for a subtotal resection of the tumor. The case presented demonstrates an example of a challenging diagnosis. Differentiating between demyelinating and neoplastic conditions is critical since the management and prognosis differ greatly. More importantly, we present a case of glioblastoma rich with Creutzfeldt-Peters cells, which has previously not been reported in the literature.

## Introduction

Creutzfeldt-Peters cells (CPC) are reactive astrocytes with fragmented nuclear inclusions. These cells are associated with demyelination and multiple sclerosis. Differentiating between multiple sclerosis and glioblastoma is usually straightforward but can be challenging in certain situations. In clinical practice, it is critical to differentiate between these two pathologies due to the vastly different management of multiple sclerosis (MS) and glioblastoma (GB). Here, we present a case of a GB that was rich with CPC and posed a diagnostic challenge. 

## Case presentation

**Figure 1:**
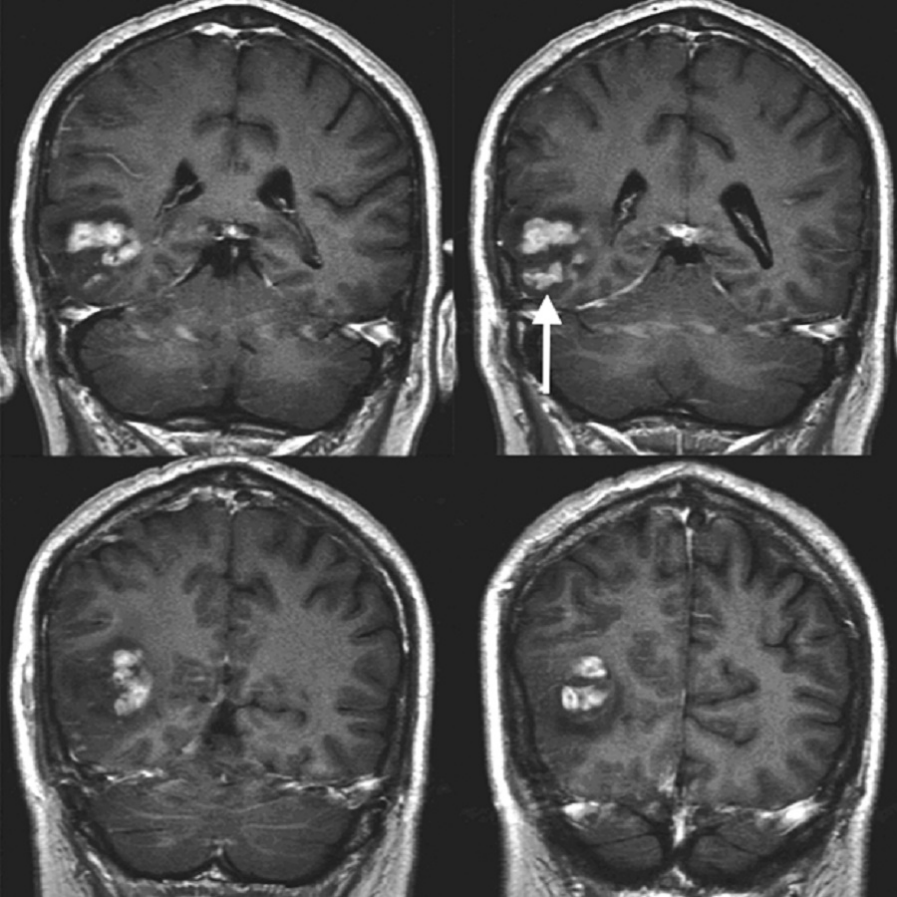
Coronal T1 post-contrast MRI Heterogeneously enhancing mass centered in the right posterior temporo-occipital region involving both the cortex and subcortical white matter with surrounding vasogenic edema (arrow) MRI: magnetic resonance imaging

We present a 78-year-old male with a previous medical history of prostate cancer, which was treated with radiation therapy, who secondarily developed urethral strictures for which he had recently undergone a urethral stricture dilatation. He presented the following day with altered mental status, nausea, and lethargy. He had no previous symptoms of headaches or other neurologic deficits. He was physically active and had otherwise been in excellent health. His family history was positive for pancreatic cancer in his father. He did not smoke or consume alcohol. On admission to the hospital, he was hyponatremic with a sodium level of 120. After fluid resuscitation, his sodium normalized to 136. A computed tomography (CT) scan revealed a mass lesion in his right posterior temporal lobe for which neurosurgery was consulted. His encephalopathy improved and his neurologic exam at the time of consultation only demonstrated a superior quadrantanopsia in his left visual field that was consistent with confrontation testing. He was placed on intravenous steroids, anticonvulsants, and an MRI scan of the brain was performed, which revealed a 4.6 x 3.1 cm mass lesion in the right posterior temporal lobe with minimal mass effect and heterogeneous contrast enhancement (Figure 1).

**Figure 2:**
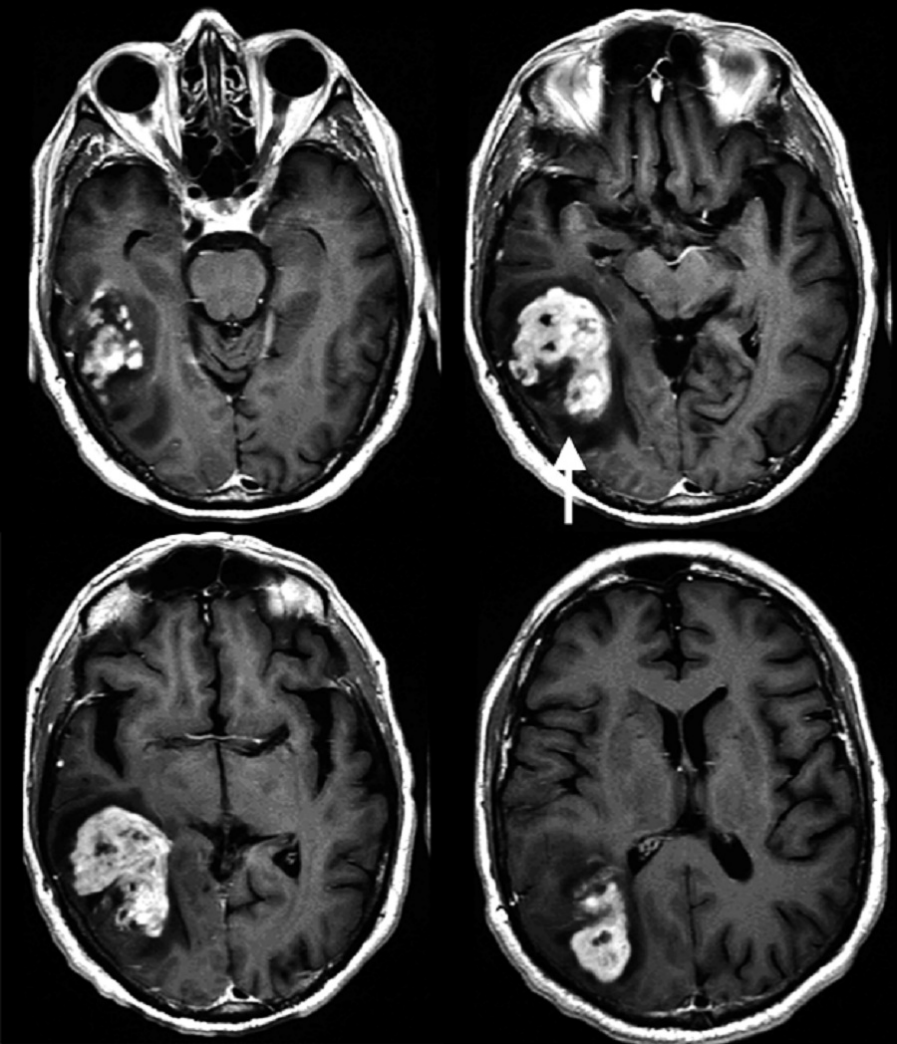
Axial T1 post-contrast MRI Interval progression of heterogeneously enhancing mass now involving the right temporal, parietal, and occipital lobes with local mass effect and surrounding vasogenic edema (arrow) MRI: magnetic resonance imaging

**Figure 3:**
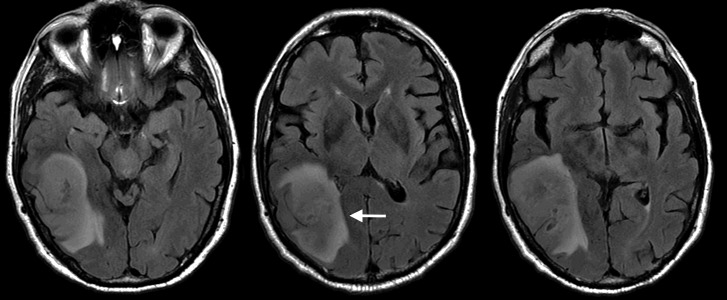
Axial T2 FLAIR MRI Minimal mass effect and fluid-attenuated inversion recovery (FLAIR) hyperintensity relative to the size of the mass (arrow) MRI: magnetic resonance imaging

After a discussion with the patient and his family, the decision was made to proceed with an open biopsy after his hematuria had resolved. The patient had a CT scan of the chest abdomen and pelvis prior to discharge, which was negative for malignancy. The patient had an outpatient follow-up appointment with repeat MRI brain three weeks later, which revealed a progression of the mass, with an interval increase in size to 5.4 x 3.4 cm, with minimal worsening of the local mass effect and vasogenic edema while on oral steroids (Figure 2 and Figure 3).

One week later, the patient underwent a craniotomy for resection of the tumor. The initial specimens were obtained and sent to pathology prior to resection. The intra-operative frozen diagnosis was read as a hypercellular lesion with rare mitotic figures, favoring inflammatory versus infectious process. The decision was then made to wait for the final diagnosis before removing the mass, and the patient was discharged home after an uneventful three days in the hospital.

**Figure 4:**
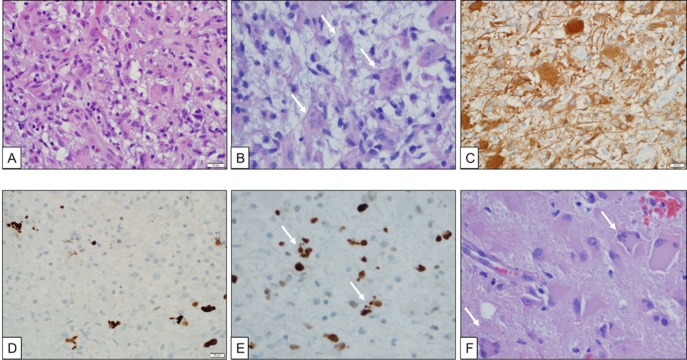
Pathology Formalin-fixed, paraffin-embedded, H&E-stained sections show hypercellular brain parenchyma with atypical astrocytes (A), macrophages, and Creutzfeldt cells (B, arrows). The atypical cells were positive for GFAP (C). Endothelial proliferation was identified with scattered fibrin thrombi but no necrosis was seen. MIB-1 was modestly elevated in tumor cells (D). P53 immunostain was positive in some cells, including the Creutzfeldt cells (E, arrows). The second surgical specimen also showed hyercellularity with atypical astrocytes and Creutzfeldt cells (F).

The histology of the lesion was unusual in that there was hypercellularity, conspicuous Creutzfeldt cells with multiple micronuclei, and abundant histiocytes on immunohistochemical evaluation (Figure 4). Vascular proliferation and fibrin thrombi were identified. The proliferation index was relatively low in certain areas. Additional immunohistochemical and molecular data were sought to achieve a diagnosis. The atypical cells were negative for the IDH1 p.R132H mutant protein and a subset of lesional cells showed expression of p53. The expression of the ATRX protein was retained. Sections were sent for further analysis with fluorescence in situ hybridization (FISH) studies, which found no evidence for PTEN loss or epidermal growth factor receptor (EGFR) amplification. The absence of alterations in either of these genes failed to provide evidence for a diagnosis of glioblastoma, World Health Organization (WHO) grade IV. Therefore, the tissue was sent for analysis with a next-generation sequencing panel covering frequently mutated regions in 50 cancer-associated genes (Ion AmpliSeq™ Cancer Hotspot Panel v2; ThermoFisher). No somatic mutations were detected in the tissue examined. Although the histologic findings are unusual, and the molecular tests performed did not reveal a specific genetic alteration, a consensus review of the histologic features by three neuropathologists led to the final diagnosis of glioblastoma, WHO grade IV, with atypical histologic features. 

**Figure 5:**
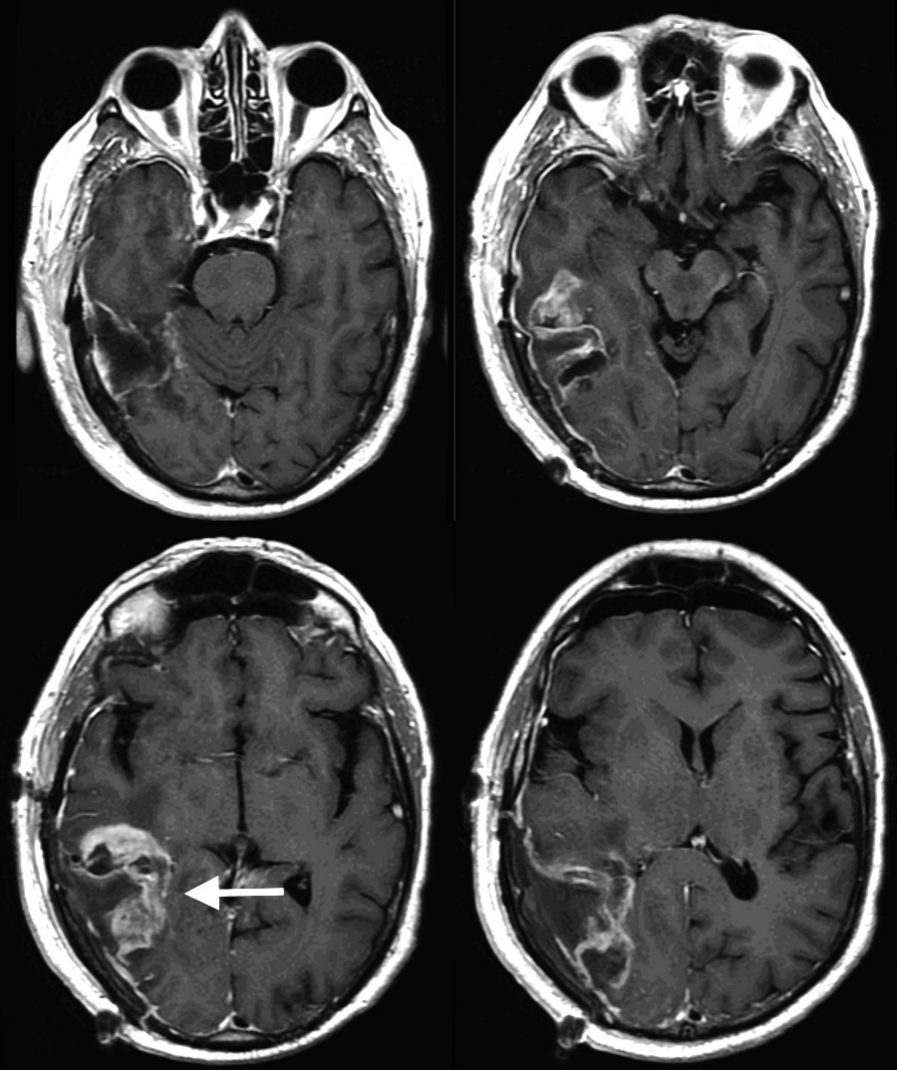
Post-operative axial T1 post-contrast MRI Post-operative surgical cavity with residual tumor anterior and medial to the resection margins (arrow) MRI: magnetic resonance imaging

The patient was taken back to the operating room approximately four weeks later for a more definitive resection of the residual tumor, which was without complication, and postoperative imaging revealed the expected subtotal resection of the tumor (Figure 5). 

The surgical specimen from the second surgical resection was also evaluated by three neuropathologists; the histologic features were similar to the initial biopsy with additional findings including cortical infiltration and subpial spread of the tumor cells, which provide additional evidence to support a diagnosis of glioblastoma, WHO grade IV.

## Discussion

Creutzfeldt-Peters cells are reactive astrocytes with fragmented nuclear inclusions (micronuclei), which are identifiable by light microscopy. These cells were first described and illustrated by Creutzfeldt in 1923. He postulated that multiple micronuclei and chromatin clumps were a byproduct of deficient karyokinesis. Peters subsequently described these cells in 1958 in the presence of acute demyelinating encephalomyelitis. The formation of micronuclei can be traced to chromosome breakage and dysfunction of the mitotic apparatus. Micronuclei consist of acentric chromosomes or chromatids or whole chromosomes/chromatids that lag behind in anaphase. The failure of micronuclei to migrate to the poles leads to their exclusion from the daughter cell during telophase [1]. Cimini et al. described that laggards cannot move to the poles due to a lack of attachment to the mitotic spindle [2]. Micronuclei within whole chromosomes contain centromeres and can be detected with fluorescence in situ hybridization, utilizing centromeric probes. Micronuclei consisting of acentric fragments do not have centromeric DNA [1].

The presence of micronuclei has been reported in a variety of neoplastic and non-neoplastic conditions. They are commonly found in cancerous cells and can serve as a marker of clastogen- or aneugen-induced chromosomal instability [3]. CPC are one such example of abnormal astrocytes with micronuclei and they are frequently found in inflammatory demyelinating conditions such as MS [4]. The critical features of MS, in particular, include: (1) perivascular mononuclear infiltrates; (2) reactive, atypical astrocytes; (3) lipid-filled macrophages; and (4) cellular heterogeneity [5]. There may be areas of remyelination in close proximity to areas of demyelination. Macrophages are the primary population and outnumber T lymphocytes by a factor of 10. Lymphocytes in MS are primarily T cell variant but B cells are also present [6].

Differentiating demyelinating from neoplastic conditions can pose a significant challenge. There are a number of reports in the literature of large ring-enhancing, space-occupying lesions that are initially considered neoplastic or infectious but on further review were deemed demyelinating. Zagzag et al. report 17 cases that appeared to be tumors but were later correctly diagnosed as demyelinating [7]. Classically, on imaging, demyelinating lesions have a C-shaped or “open-ring” pattern of enhancement. This is in contrast to neoplastic or infectious etiology, which has a complete ring of enhancement [8]. Masdeu and colleagues conducted a study to confirm these patterns. There were three groups (demyelinating, infectious, and tumor), with 32 confirmed cases each. The open ring sign was present in 70% of the 32 cases of demyelinating diseases. This is in comparison to one of 32 in the neoplastic group and zero of 32 in the infectious group [9].

On histology, there are several factors that can lead to confusion between demyelinating and neoplastic conditions. Five such patterns were identified by Zagzag et al., which include hypercellularity, the presence of pleomorphic reactive astrocytes with bizarre nuclei, the presence of mitotic figures, necrosis, and areas of cystic degeneration or cavitation in edematous tissues [7]. Erana-Rojas et al. noted that prominent-appearing reactive astrocytes and easily identifiable mitotic figures of astrocytes and macrophages can add to the confusion and appear similar to diffuse astrocytoma or oligodendroglioma [8]. To avoid misdiagnosis, there are features on histology that can lead to the correct diagnosis of demyelination. Unlike tumors, demyelinating conditions tend to have well-defined borders and lack vascular hyperplasia. Additionally, demyelinating conditions have a prominent inflammatory component, which is a mix of lymphocytes and plasma cells with foamy macrophages [7].

The case presented above is an example of a challenging clinical, radiographic, and histologic diagnosis. A circular contrast enhancement pattern on the MRI would support a neoplastic or infectious etiology. Histologically though, CPC and granular mitoses would suggest a demyelinating disease. In the literature, there is one reported case of a GB with CPC present. The patient underwent a stereotactic biopsy, which demonstrated reactive astrocytes, including CPC and lipid-laden macrophages, consistent with MS. Deeper sections from the same initial biopsy block demonstrated a lesion that was a brain-invasive astrocytoma with hypercellularity and frequent mitoses. The patient underwent resection and the specimen was consistent with GB without any sign of demyelination. The authors of this case report postulated that demyelination of the surrounding tissue was a result of the breakdown of the adjacent blood-brain barrier and subsequent inflammation due to an antigen-rich environment [10]. Our case is unique in the sense that the center of the lesion demonstrated CPC. Hence, it is unlikely that a similar mechanism exists in the previously described case report.

## Conclusions

Differentiating demyelinating and neoplastic conditions can pose a challenge in clinical practice. Clinical, radiographic, and histologic features help guide clinical practitioners to the appropriate diagnosis. Our case illustrates how tried and true guidelines may not always lead to the correct answer.
